# Folate and global health umbrella review series, part 1: methodological framework and syntheses on anaemia and neural tube defects

**DOI:** 10.7189/jogh.16.04014

**Published:** 2026-01-30

**Authors:** Samantha Yoo, Azita Montazeri, Derrick Bennett, Yacong Bo, Peizhan Chen, Susan Duthie, Natalie Jensen, Atipatsa Kaminga, Jun-Shi Lai, Xue Li, Amanda J MacFarlane, Homero Martinez, Helene McNulty, Franco Momoli, Peter Mossey, Patrick Mullie, Ron Munger, Rajendra Prasad Parajuli, Monique Potvin Kent, Michele Rubini, Marjanne Senekal, Lindsey Sikora, Alain Stintzi, Evropi Theodoratou, Hui Wang, Chittaranjan Yajnik, Ann Yaktine, Julian Little

**Affiliations:** 1 School of Epidemiology and Public Health, Faculty of Medicine, University of Ottawa; 2 Nuffield Department of Population Health, University of Oxford, UK; 3 School of Public Health, Xinxiang Medical University, China; 4 Clinical Research Centre, Ruijin Hospital, Shanghai Jiao Tong University School of Medicine, Shanghai, China; 5 School of Pharmacy and Life Sciences, Robert Gordon University, Scotland, UK; 6 Department of Mathematics and Statistics, Faculty of Science, Technology and Innovation, Mzuzu University, Malawi; 7 Singapore Institute for Clinical Sciences, Agency for Science, Technology and Research, Singapore; 8 Centre for Population Health Sciences, University of Edinburgh, UK; 9 Nutrition Research Division, Health Canada, Canada; 10 Research and Development Unit, Nutrition International, Canada; 11 Nutrition Innovation Centre for Food and Health, School of Biomedical Sciences, Ulster University, Coleraine, Northern Ireland, UK; 12 School of Dentistry, University of Dundee, Scotland, UK; 13 International Prevention Research Institute, Lyon, France; 14 Department of Nutrition, Dietetics, and Food Sciences, College of Agriculture and Applied Sciences, Utah State University, USA; 15 Herbert Wertheim School of Public Health and Human Longevity Science, University of California San Diego, USA; 16 Department of Neuroscience and Rehabilitation, University of Ferrara, Italy; 17 Department of Human Biology, Faculty of Health Sciences, University of Cape Town, South Africa; 18 Health Sciences Library, University of Ottawa, Canada; 19 School of Pharmacy, Faculty of Medicine, University of Ottawa, Canada; 20 Centre for Global Health, Usher Institute, College of Medicine and Veterinary Medicine, University of Edinburgh, UK; 21 School of Public Health, Faculty of Medicine, Shanghai Jiao Tong University, China; 22 Diabetes Unit, KEM Hospital Research Centre, India; 23 Food and Nutrition Board, Health and Medicine Division, National Academies of Sciences, Engineering, and Medicine, USA

## Abstract

**Background:**

Folate is essential for normal growth and in human health throughout the lifecycle. Clinical deficiency of folate impairs DNA synthesis and results in megaloblastic anaemia, while suboptimal folate status before and in early pregnancy results in an elevated risk of neural tube defects (NTD). The evidence on the association of folate status with other health outcomes is largely fragmented and understudied. We conducted a series of umbrella reviews examining the association between folate and multiple health outcomes in various populations and settings.

**Methods:**

We searched MEDLINE, Embase, CINAHL, the Cochrane Library, and DARE from inception to February 2024 for systematic reviews with or without meta-analyses examining an association between folate intake/status and any health outcome. We performed screening and data extraction in duplicate and assessed the risk of bias using the ROBIS tool. Evidence was then characterised into unique associations (unique exposure measure – unique outcome measure – unique setting). For each category of unique associations, we identified the evidence based on the statistical power, recency of publication and the potential risk of bias. All unique associations were evaluated for credibility using predefined criteria.

**Results:**

We retrieved 3565 records and included 283 in the final synthesis. The evidence on anaemia consisted of four intervention trials demonstrating effectiveness of folic acid supplementation during pregnancy in reducing the risk of megaloblastic anaemia (relative risk (RR) = 0.21; 95% CI = 0.11, 0.38; *I*^2^ = 15%). Maternal folic acid use was also significantly inversely related to the prevention of NTD at birth (RR = 0.31; 95% CI = 0.16, 0.60; *I*^2^ = 0%) and NTD recurrence (RR = 0.30; 95% CI = 0.14, 0.65; *I*^2^ = 0%). This relationship was supported by the inverse association reported between low maternal blood folate concentrations and the increased risk of NTD. Further evidence showed that fortification of food with folic acid was associated with the lower prevalence of NTD on a population-level.

**Conclusion:**

In NTDs and anaemia, we identified strong evidence supporting the protective role of folate status based on intervention trials and observational studies. More recent reviews examining the role of folate in other less well understood health conditions will be presented in the subsequent reports.

**Registration:**

PROSPERO: CRD42021265041.

Folate is required for one-carbon metabolism, a network of reactions involving the transfer and utilisation of one-carbon units for key biological reactions, including the biosynthesis of DNA and RNA, amino acid metabolism, and methylation processes [1]. It is thus essential for normal growth and development and in human health throughout the lifecycle. Clinical deficiency of folate impairs DNA synthesis and nuclear division and results in megaloblastic anaemia [2,3], a condition characterised by abnormally enlarged blood cell precursors produced in the bone marrow and macrocytes in the peripheral blood. The population-level thresholds for folate deficiency, based on the risk for megaloblastic anaemia, are serum total folate concentration <6.8 ng/mL or red blood cell (RBC) folate concentration <226.5 nmol/L [4] based on microbiological assays.

Apart from these cut-offs for clinical deficiency, other folate status thresholds have been associated with a risk for other health outcomes. Importantly, suboptimal folate status in pregnancy results in an elevated risk of neural tube defects (NTD), with one study showing that the risk of having a child with an NTD was very strongly inversely related to the mother’s RBC folate concentration, and that the risk remained high even when maternal RBC was well above the folate-deficiency cut-point [5]. Thus, suboptimal RBC folate was set at <906 nmol/L, the maternal level above which no further benefit on NTD risk was evident [6]. Policy initiatives, namely fortification of food with folic acid (*i.e.* a synthetic form of folate) and folic acid supplementation for women of reproductive age [7], were implemented following the publication of conclusive evidence in the form of randomised controlled trials (RCTs) showing that intervention with folic acid in women before and in early pregnancy could prevent both first occurrence [8] and recurrence of NTD [9]. Mandatory fortification of staple grains – now in places in over 90 countries worldwide – has achieved substantial reduction in the incidence of NTD over the past three decades [10,11]. However, the evidence on the association of folate status with other health outcomes, particularly non-communicable diseases, is largely fragmented and/or understudied. The role of folate in health across the lifespan calls for a comprehensive synthesis of epidemiological evidence.

In a series of umbrella reviews entitled ‘Folate and Global Health’, we sought to integrate the existing evidence on the associations between folate and health effects, including, but not limited to, NTDs and anaemia, and critically appraise their methodological quality and credibility. Umbrella reviews allow for the identification, triangulation, and assessment of the volume and quality of systematic reviews and meta-analyses available on a given topic to date [12,13].

The most recent umbrella review of folate and health outcomes was conducted in 2018 [14], but was limited to meta-analyses of intervention trials and prospective and retrospective observational studies and to populations aged ≥18 years. We engaged its authors to update and expand its scope to systematic reviews without meta-analyses and to foetal, neonatal, and adolescent populations. We further separated the folate exposure measures by type.

Our findings will be presented in a subsequent series of six reports organised by the following health outcomes: anaemia and NTDs; cancers; cardiovascular, cerebrovascular, and metabolic disorders; autoimmune and skeletal outcomes; neuropsyhicatric disorders; pregnancy and maternal/offspring outcomes Here, we aimed to detail the methodologies used in this umbrella review series; summarise the volume of evidence identified across all of the health outcomes; and present our findings on the causal and preventive role of folate on anaemia and NTDs.

## METHODS

Here, we describe the methodological approach used throughout the syntheses in the series. A common set of search strategies, eligibility criteria, and appraisal considerations was applied consistently. Any additional methodological considerations or modifications from the reference approach will be separately reported in each relevant subsequent synthesis.

### Data sources and search

We conducted a series of umbrella reviews of systematic reviews and meta-analyses that investigated the associations of folate, measured as dietary intake, supplementation, or blood concentrations, with any health outcome. A health science librarian (LS) searcher MEDLINE and MEDLINE in Process *via* Ovid (1946 to 13 February 2024), Embase Classic + Embase *via* Ovid (1947 to 13 February 2024), CINAHL *via* EBSCOHost (1981 to 13 February 2024), the Cochrane Database of Systematic Reviews (2005 to 28 December 2021), and the Database of Abstracts of Reviews of Effects (DARE) *via* Ovid (1994 to first quarter of 2016) (Table S1 in the **Online Supplementary Document**). We were unable to extend the search to CNKI and Wanfang, as described in the initial protocol, due to resource constraints.

### Eligibility criteria

We included systematic reviews with or without meta-analyses that examined RCTs, non-randomised intervention trials, prospective or retrospective cohort studies, case-control studies, or cross-sectional studies, provided they examined the associations of any measure of folate status (dietary intake, supplementation, or biomarkers) with any health outcome were eligible. We excluded case reports, case series, commentaries, protocols, or scoping reviews; reviews that investigated circulating homocysteine as a marker of folate status, multivitamins or multiple nutrients without assessment of the independent effect of folate, the prevalence of folate inadequacy, or health outcomes without clear definitions/diagnostic criteria; and reviews that reported on a single study for a given outcome. We set no restrictions on the study population, language, or date of publication.

### Data extraction

Two reviewers (SY, AM, or NJ) independently screened the articles in two stages (first by title and abstract and then by full text). All discrepancies were resolved by discussion between the two reviewers. The following data were extracted in duplicate (SY, AM) using a standardised template: study information (first author, year of publication, year of search, exposure measure, outcome measure, risk of bias assessment), study population (eligibility criteria, countries represented in the review, participants’ age, sex, other sociodemographic features, if any), exposure details (type of exposure measure, method of measurement, time of measurement), outcome details (definition of outcome, measurement tool/scale used), qualitative synthesis, and quantitative data (number of primary studies, number of total participants, number of cases, reported summary effect, heterogeneity measure, measure of small study effects, methodological quality assessment, dose-response effects, subgroup analyses, if available). Discrepancies were resolved through discussion.

### Assessment of credibility

We assessed the credibility of evidence for all unique associations using a predefined set of credibility criteria (**Table 1**), which is widely used in umbrella reviews [15,16]. For positive associations (*P* < 0.05), we ranked the evidence into four classes: convincing, highly suggestive, suggestive, and weak; for non-significant associations (*P* > 0.05), we formed two classes: suggestive and weak.

**Table 1:** Criteria for credibility assessment

Category	Associations
Directional associations	
*Convincing*	With statistical significance of *P* < 10^−6^
	Based on ˃1000 cases (or ˃20 000 participants for continuous outcomes)
	For which largest component study reports a statistically significant result (*P* < 0.05) and has a 95% prediction interval that excludes the null
	Which do not have large heterogeneity (*I*^2^<50%)
	Show no evidence of small study effects (*P* ˃ 0.10) or of excess significance bias (*P* ˃ 0.10)
*Highly suggestive*	With statistical significance of *P* < 10^−6^
	Based on ˃1000 cases (or ˃20000 participants for continuous outcomes)
	For which largest component study reports a statistically significant result (*P* < 0.05)
*Suggestive*	With statistical significance of *P* < 0.01
	Based on ˃1000 cases (or ˃20 000 participants for continuous outcomes)
*Weak*	With statistical significance of *P* < 0.05
Null associations	
*Suggestive*	Based on ˃1000 cases (or ˃20 000 participants for continuous outcomes);
	Which do not have large heterogeneity (*I*^2^<50%)
	With statistical significance of *P* ˃ 0.10
*Weak*	With statistical significance of 0.05 < *P* < 0.10

### Synthesis

We first grouped the reviews into 11 broad health outcome categories: cancers, cardiovascular outcomes, cognitive function, congenital anomalies, pregnancy-related outcomes, maternal-offspring outcomes (other than congenital anomalies), metabolic conditions, skeletal outcomes, neuropsychiatric outcomes, infectious diseases, and others. We further categorised a review within a given a health outcome category by types of exposure measures. Any syntheses limited to specific population groups (*e.g.* age group, country, *etc*.) were treated as unique. For each unique exposure measure – unique outcome measure – unique setting examination (hereinafter referred to as ‘unique associations’), we critically examined the collected evidence and the reported summary effects. If the summary effects were concordant in direction, magnitude, and statistical significance, we identified the evidence with the largest number of total participants. If discordant, we identified the evidence the largest number of total participants included in the synthesis, the largest number of cases (for binary outcomes), the recency of publication, and the highest methodological quality as assessed by the ROBIS tool [17].

For unique associations that were assessed to be of a highly suggestive level of certainty, we made best efforts, depending on the data availability, to re-calculate the summary effects and 95% confidence intervals (CIs); predictive intervals to understand the dispersion of effect sizes [18]; heterogeneity between the studies using *I*^2^ statistics and *P*-values; small study effects using Egger’s test of symmetry [19] with a significance threshold *P* < 0.10; and excessive significance [20] with a threshold *P* < 0.10.

Lastly, we conducted sensitivity analyses in cases where the evidence selected for a unique association consisted entirely or predominantly of retrospective studies. We selected evidence in the same category comprising entirely or predominantly prospective investigations (intervention trials, prospective cohort studies, nested case-control studies, or case cohort studies) and compared the findings to see if retrospective design of component studies biased the pooled estimates in any direction. If evidence comprising prospective studies was not identified in a given category, we reported the absence of such evidence.

### Risk of bias assessment

Two reviewers (AM, NJ) independently assessed the quality of the syntheses using the ROBIS tool, with conflicts in resolved through discussion. The ROBIS allows for an assessment of risk of bias across four domains – study eligibility criteria, identification and selection of studies, data collection and study appraisal, and synthesis and findings – as well as the overall risk of bias in the interpretation of review outcomes. We did not generate a final score for each article in accordance with the authors’ recommendations; instead, we presented a descriptive summary of the levels of risk of bias across all domains for each article included for analysis.

## RESULTS

### Overview of search results

**Figure 1:**
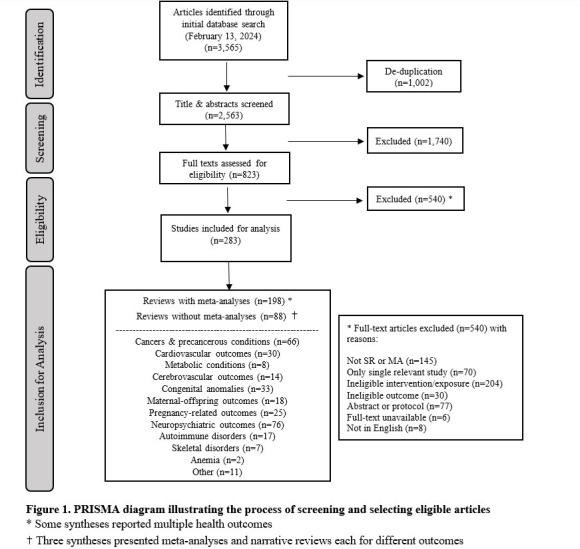
PRISMA diagram illustrating the process of screening and selecting eligible articles. *Some syntheses reported multiple health outcomes. †Three syntheses presented meta-analyses and narrative reviews each for different outcomes.

Our search retrieved 3565 records. After de-duplication and first stage screening based on titles and abstracts, the full texts of 825 articles remained for review, of which 538 were excluded for not having the design of a systematic review (n = 145 articles); investigating exposures to multiple vitamins without specifying the exposure to folate (n = 204); and having a single eligible component study in the evidence synthesis (n = 69). This left 287 articles for inclusion in our synthesis (**Figure 1**).

The evidence included in our review reported on 12 broad health outcomes: cancers and precancerous lesions (n = 66), cardiovascular outcomes (n = 30), cerebrovascular outcomes (n = 13), NTD (n = 15), congenital anomalies other than NTD (n = 18), pregnancy-related outcomes (n = 24), maternal-offspring outcomes (n = 17), neuropsychiatric outcomes (n = 79), autoimmune conditions (n = 17), metabolic outcomes (n = 9), skeletal outcomes (n = 6), anaemia (n = 2), and other outcomes (n = 11).

**Figure 2:**
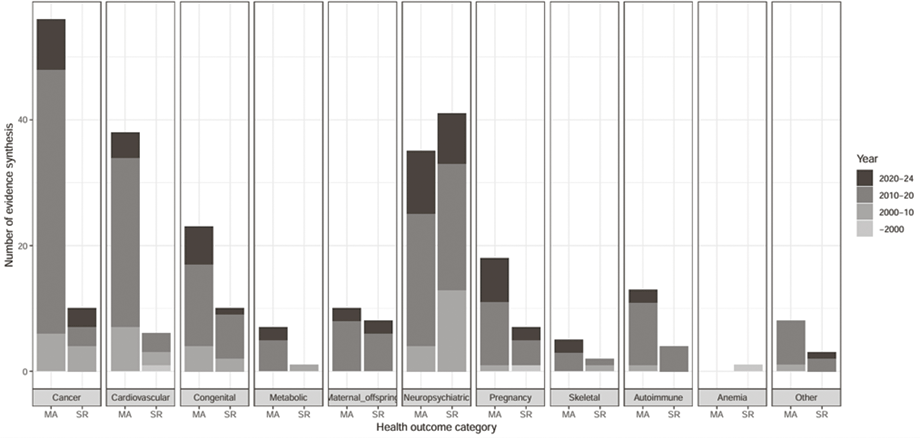
Distribution of evidence syntheses identified across the health outcomes and year.

More than half of the evidence was published after 2010 (64.0%) and a fifth after 2020 (19.2%). The syntheses were predominantly quantitative (as opposed to narrative) for all outcomes, except for neuropsychiatric disorders. Neuropsychiatric, cancer, and cardiovascular outcomes accounted for the largest proportion of the evidence, and were represented in 78, 68, 30 articles, respectively (**Figure 2**).

### Anaemia

Two syntheses of intervention trials [21,22] investigated the relationship between folic acid supplementation and anaemia, reporting six unique associations: haemoglobin concentration among pregnant women and non-pregnant women; megaloblastic anaemia among pregnant women and premature infants; pre-delivery anaemia among pregnant women; and pre-delivery haemoglobin concentration among pregnant women (Table S2a in the **Online Supplementary Document**). Quantitative syntheses were provided for three of these unique associations.

Among pregnant women, use of folic acid supplement at 0.01-5.0 mg/d significantly reduced the development of megaloblastic anaemia (RR = 0.21; 95% CI = 0.11, 0.38), *I*^2^ = 15%) [21]. However, the effects of folic acid on the mean haemoglobin concentration (MD = −0.03 g/dL; 95% CI = -0.25, 0.19; *I*^2^ = 95%) or prevention of anaemia among pregnant women (RR = 0.62; 95% CI = 0.35, 1.10; *I*^2^ = 90%) were heterogeneous. The doses of folic acid used for investigation of mean haemoglobin concentration and prevention of anaemia were 0.05–350 mg/d and 0.45–5.0 mg/d, respectively, and durations were not reported (**Table 2**).

**Table 2:** Summary of the meta-analyses reporting the association of assignment to take folic acid supplement and the risk of anaemia

Author (year)	Exposure (dose, duration)	Outcome	Country/region	Number of studies and their design	Total number (number of cases)	Comparator	Summary effect (g/dL)	*I*^2^ (*P*-value)
Lassi *et al*. (2013) [21]	FA supplement (0.45–5 mg/d)	Pre-delivery anaemia	UK, Nigeria, India, Myanmar, Australia	8	4149 (577)	None or placebo or other nutrients	RR = 0.62 (95% CI = 0.35, 1.10)	90% (<0.001)
	FA supplement (0.05–350 mg/d)	Pre-delivery haemoglobin	UK, Switzerland, France, Finland, Chile, South Africa, Nigeria, Thailand	12	1806 (NR)	None or placebo or other nutrients	MD = −0.03 (95% CI = −0.25, 0.19)	95% (<0.001)
	FA supplement (0.01–5mg/d)	Megaloblastic anaemia	UK, Nigeria	4	3839 (89)	None or placebo or other nutrients	RR = 0.21 (95% CI = 0.11, 0.38)	15% (0.32)

CI – confidence interval, FA – folic acid, MD – mean difference, NR – not reported, RCT – randomised controlled trial, RR – relative risk

The narrative synthesis [22] reported similar findings: folic acid supplementation was effective in prevention of megaloblastic anaemia among pregnant women (two trials, 346 participants, folic acid 0.3–1.0 mg/d for 4–16 weeks) and premature/low birthweight infants (five trials, 700 participants, folic acid 0.05–0.1 mg/d for 4 weeks to 12 months), while the effect on haemoglobin concentration was unclear among pregnant (11 trials, 3993 participants, folic acid 0.5-5.0 mg/d for 4–24 weeks) or non-pregnant women (four trials, 1053 participants, folic acid 1–15 mg/d for 5–22 weeks).

All of the identified evidence on haematological parameters was based on intervention trials, so we did not perforfm any sensitivity analysis.

### NTD

We identified 15 syntheses (11 with meta-analyses and 4 without meta-analyses) that examined the relationship of folate status with NTD (Table S2b in the **Online Supplementary Document**). They investigated four associations: maternal supplement intake of folic acid before or during pregnancy and prevalence of NTD at birth (seven studies) [23–29]; folic acid fortification and prevalence of NTD at birth (three studies) [25,30,31]; maternal serum/ plasma/ RBC folate concentration and prevalence of NTD at birth (two studies) [32,33]; and maternal supplement intake of folic acid before or during pregnancy and recurrence of NTD in women with history of NTD in previous pregnancies (two studies) [25,29]. Two studies [23,34] were updates of previously published reviews [24,35].

Of the 11 meta-analyses, eight examined specific comparators, countries/regions, ethnicities, or study designs (Table S3 in the **Online Supplementary Document**). A total of 28 unique associations were identified (**Table 3**): maternal use of folic acid supplement (with or without other nutrients) and NTD prevalence at birth using different comparators (none, placebo, other nutrients, or other nutrients included in the intervention group) or in different countries/regions; folic acid fortification at the population-level and the prevalence at birth of total NTD, spina bifida, anencephaly, cephalocele in general or in low- and middle-income countries (LMICs); maternal RBC/plasma/serum folate concentrations and NTD prevalence at birth or NTD recurrence (sub-grouped by component study design and ethnicity).

**Table 3:** Summary of the identified meta-analyses reporting the association of folate intake/status with the risk of neural tube defects

Author (year)	Exposure (dose, duration)	Country/region	Number of studies and their design	Total number (number of cases)			Comparator	Summary effect		*I*^2^ (*P*-value)	*P*-value*	
**Prevalence of NTD at birth**												
De-Regil *et al*. (2010) [24]	Maternal supplement (FA only; 0.36–4.0 mg/d)	Ireland, UK	2 intervention trials	299 (9)			None or placebo	RR = 0.32 (95% CI = 0.08, 1.34)		0% (0.60)		
Blencowe *et al*. (2010) [25]	Maternal supplement (0.36 mg/d or 5.0 mg/week)	Hungary, China	1 RCT, 3 PC	NR (NR)			None or placebo	RR = 0.38 (95% CI = 0.29, 0.51)		27.9% (0.24)		
De-Regil *et al*. (2015) [23]	Maternal supplement (FA with other nutrients *vs*. other nutrients; 0.36–4.0 mg/d)	Hungary, Israel, Australia, Canada, Russia, France, UK, Ireland, India	4 intervention trials	6512 (49)			Other micronutrients	RR = 0.31 (95% CI = 0.16, 0.60)		0% (0.74)		
De-Regil *et al*. (2015) [23]	Maternal supplement (FA with other nutrients *vs*. same other nutrients; 0.36–4.0 mg/d)	Ireland, UK, Israel, Australia, Canada, Russia, France	2 intervention trials	1371 (28)			Same other nutrients	RR = 0.29 (95% CI = 0.12, 0.70)		0% (0.92)		
Bitwew *et al*. (2020) [26]	Maternal supplement	Ethiopia	4 CC	1592 (418)			Mothers of unaffected infants	OR = 0.32 (95% CI = 0.17, 0.60)		36% (0.19)		
Atlaw *et al*. (2021) [27]	Maternal supplement (NR)	Africa (Egypt, Tunisia, Ethiopia, Algeria)	6 CC, 1 CS	1963 (463)			Mothers of unaffected infants	OR = 0.4 (95% CI = 0.19, 0.85)		78% (0.001)		
Lassi *et al*. (2021) [28]	Maternal supplement (0.4–5.0 mg/d)	China, Honduras, Brazil, Cuba	2 intervention trials	248 056 (130 243)			Placebo	RR = 0.53 (95% CI = 0.41, 0.67)		0% (0.36)		
Tang *et al*. (2015) [32]	Plasma/serum folate	See below	14 CC, 15 PC	5384 (1,694)			Mothers of unaffected infants	RoM = 0.93 (95% CI = 0.88, 0.97)		73%	0.05	
		Africa, Netherlands, Britain, Mexico, Canada, Brazil, America, India, Egypt, Norway	14 (CC)	2260 (825)				RoM = 0.95 (95% CI = 0.86, 1.04)		84%		
		Netherlands, China, Ireland, America, Turkey, Iran, Finland, Britain, Canada, Egypt	15 (PC)	3124 (869)				RoM = 0.91 (95% CI = 0.86, 0.96)		40%		
		NR	Asian (NR)	1790 (699)				RoM = 0.88 (95% CI = 0.81, 0.90)		2%		
		NR	Caucasian (NR)†	2813 (666)				RoM = 0.93 (95% CI = 0.88, 0.99)		67%		
Tang *et al*. (2015) [32]	RBC folate	See below	24 (10 CC, 14 PC)	1455 (628)			Mothers of unaffected infants	RoM = 0.92 (95% CI = 0.86, 0.98)		72%	0.22	
		Africa, Netherlands, Britain, Mexico, Canada, Norway, India, America	10 CC	848 (414)				RoM = 0.91 (95% CI = 0.84, 0.98)		74%		
		Netherlands, Ireland, Mexico, UK	14 PC	607 (214)				RoM = 0.95 (95% CI = 0.80, 1.14)		67%		
		NR	Asian (NR)	59 (35)				RoM = 0.74 (95% CI = 0.55, 0.98)		NA		
		NR	Caucasian (NR)†	1032 (405)				RoM = 0.90 (95% CI = 0.82, 0.98)		78%		
Yadav *et al*. (2021) [33]	Serum/RBC folate	NR	36 CC	6114 (2131)			Mothers of unaffected infants	SMD = −0.48 (95% CI = −0.77, −0.19)		95.73% (<0.001)	>0.05	
			Asian (NR)	Asian (NR)				SMD = −1.37 (95% CI = −2.41, −0.61)		97.85% (<0.001)		
			Caucasian (NR)†	Caucasian (NR)†				SMD = −0.17 (95% CI = −0.35, 0.004)		78.89% (<0.001)		
			African (NR)	African (NR)				SMD = −0.03 (95% CI = −0.56, 0.49)		60.89% (0.11)		
Blencowe *et al*. (2010) [25]	Fortification (NR)	Hungary, China	8 Before-after	NR (NR)			Pre- *vs*. post-fortification	RR = 0.54 (95% CI = 0.46, 0.63)		69.2% (0.002)		
Keats *et al*. (2019) [30]	Fortification (wheat 1.5–5.0 mg/kg; maize 1.3–2.2 mg/kg)	LMICs‡	17 mixed§	19 816 008 (13 494)			Pre- *vs*. post-fortification	OR = 0.59 (95% CI = 0.49, 0.70)		84% (<0.001)		
**Spina bifida**												
Atta *et al*. (2016) [31]	Fortification (NR)	NR	123 before-after		NR (NR)		Mandatory vs no fortification		Values presented per 100 000 population. Among live births, 33.86 (95% CI = 31.05, 36.92) among mandatory fortification countries *vs*. 48.35 (95% CI = 41.07, 56.93) among voluntary fortification or no fortification countries. Among live births and stillbirths, 30.37 per 100 000 (95% CI = 27.42, 33.63) mandatory fortification countries *vs*. 47.74 (95% CI = 43.66, 52.20) among voluntary fortification or no fortification countries. Among live births, still births, terminated pregnancies, 35.22 (95% CI = 32.18, 38.56) among voluntary fortification *vs*. 52.29 (95% CI = 46.28, 59.08) among no fortification countries.			
Keats *et al*. (2019) [30]	Fortification (wheat 1.5–5.0 mg/kg; maize 1.3–2.2 mg/kg)	LMICs‡	9 mixed§		21 175 429 (6385)				OR = 0.66 (95% CI = 0.53, 0.82)	88% (0.00001)		
**Anencephaly**												
Keats *et al*. (2019) [30]	Fortification (wheat 1.5–5.0 mg/kg; maize 1.3–2.2 mg/kg), anencephaly	LMICs‡	9 mixed§		21 174 429 (6 876)			OR = 0.49 (95% CI = 0.40, 0.60)		78% (<0.0001)		
**Encephalocele**												
Keats *et al*. (2019) [30]	Fortification (wheat 1.5–5.0 mg/kg; maize 1.3–2.2 mg/kg)	LMICs‡	8 mixed§		21 049 821 (1857)			OR = 0.64 (95% CI = 0.47, 0.88)		75% (0.0003)		
**NTD recurrence**												
Blencowe *et al*. (2010) [25]	Maternal supplement (0.36 mg/d or 5 mg/week)	UK, Ireland, Hungary, Australia, Italy, France, Canada, Russia	3 RCT	NR (NR)			None or placebo	RR = 0.30 (95% CI = 0.14, 0.65)		0% (0.87)		

CC – case-control study, CCoh – case cohort, CI – confidence interval, CS – cross-sectional, LMIC – low- and middle-income country, NCC – nested case-control, NR – not reported, OR – odds ratio, PC – prospective cohort, RBC – red blood cell, RCT – randomised controlled trial, RoM – ratio of means, RR – risk ratio

*Egger’s test.

†‘Caucasian’ is considered an imprecise and outdated terminology which is commonly used to describe individuals of European descent.

‡Fiji, Cameroon, Iran, Brazil, South Africa, Peru, Costa Rica, Argentina, Tanzania, Jordan, China.

§Combination of multiple study designs (hospital-based surveillance, repeated cross-sectional, before-after studies, retrospective administrative records).

The reported associations between maternal folic acid supplementation and NTD prevalence at birth were comparable in direction and magnitude, with relative risks (RRs) ranging from 0.29 (95% CI = 0.12, 0.70) to 0.53 (95% CI = 0.41, 0.67). The syntheses published between 2010 and 2015 pooled from intervention trials conducted predominantly in Europe and North America reported strong inverse associations, with RRs ranging from 0.29 (95% CI = 0.12, 0.70) to 0.38 (95% CI = 0.29, 0.51), and *I*^2^ values from 0% to 27.9% [23–25]. Two reviews published after 2020 [26,27] synthesised retrospective studies conducted in Africa reported comparable associations, with RRs ranging from 0.32 (95% CI = 0.17, 0.60) to 0.4 (95% CI = 0.19, 0.85), and *I*^2^ values from 36% to 78%. One synthesis [28] pooled two community-based intervention trials in China and South America and reported an RR of 0.53 (95% CI = 0.41, 0.67; *I*^2^ = 0%). The doses used in the primary intervention trials ranged from 0.36 mg/d to 5.0 mg/d for all syntheses that reported them. Information on the duration was frequently missing.

The relationship between maternal blood folate concentration and the prevalence at birth of NTD was examined in two meta-analyses of observational studies. Tang *et al*. [32] reported a marginally lower concentration of folate in plasma of mothers affected by NTD compared to mothers not affected by NTD (ratio of means (RoM) = 0.93; 95% CI = 0.88, 0.97; *I*^2^ = 73%). This inverse association remained significant in the author-identified subgroups of prospective studies, and individuals of Asian and ‘Caucasian’ ethnicity. Investigations of RBC folate concentration produced comparable results (RoM = 0.92; 95% CI = 0.86, 0.98; *I*^2^ = 72%) and replicated in the same three subgroups. Pooling serum and RBC folate concentrations together and limiting their analyses to case-control studies, Yadav *et al*. [33] showed that mothers affected by NTD had significantly lower folate concentrations compared to non-affected mothers (SMD = −0.48; 95% CI = −0.77, −0.19, *I*^2^ = 95.73%).

The evidence on maternal folic acid supplementation and prevention of NTD recurrence pooled three RCTs conducted in women with a history of NTD-affected pregnancies and reported strong inverse association (RR = 0.30; 95% CI = 0.14, 0.65; *I*^2^ = 0%). The doses of folic acid used in the primary studies ranged from 0.36 to 1 mg/d.

Narrative syntheses examining the association between maternal supplementation with folic acid and the prevalence of NTD at birth reported similar findings. Doses of folic acid used in the fortification were not reported.

Three meta-analyses assessed the correlation between folic acid fortification and the prevalence of NTD at birth at a population-level using before-after studies, hospital-based surveillance, or health administrative records. The effects on total NTD prevalence at birth in different countries after mandatory large-scale food fortification with folic acid were comparable (RR = 0.54 (95% CI = 0.46, 0.63), *I*^2^ = 69.2%; OR = 0.59 (95% CI = 0.49, 0.70), *I*^2^ = 84%) [25,30]. A focused synthesis on the prevalence of spina bifida at birth, comparing countries with vs without mandatory folic acid fortification showed significantly lower prevalence of spina bifida in mandatory fortification countries across different birth cohorts [31]. The effects on the prevalence at birth of spina bifida, anencephaly, and encephalocele were assessed in LMICs, showing largely comparable magnitudes of inverse associations (OR = 0.66 (95% CI = 0.53, 0.82), *I*^2^ = 84%; OR = 0.49 (95% CI = 0.40, 0.60), *I*^2^ = 88%; OR = 0.64 (0.47, 0.88), *I*^2^ = 75%, respectively) [30]. Doses of fortification were reported only in the evidence examining LMICs (1.5-5.0 mg/kg of wheat and 1.3-2.2 mg/kg of maize).

The evidence on the relationship between maternal supplementation of folic acid before or during pregnancy and prevalence of NTD at birth or recurrence was largely based on intervention trials [23–25,28,29]. Two unique associations focusing specifically on four countries in Africa pooled estimates entirely from retrospective studies; however, we did not identify an alternative synthesis for these associations for prospective studies. Evidence on the relationships between maternal biomarkers and the prevalence of NTD at birth also came from case-control studies; however, separate sub-analyses were reported by design of the component studies (**Table 3**).

### Credibility assessment

The credibility of evidence on the relationship between folate status and anaemia and NTDs was considered in a contextualised manner rather than by applying the commonly used criteria [15,16]. Key factors considered were the timeline of evidence development in folate research, rigor of the primary studies that contributed to the evidence, causal relationship demonstrated from the folic acid interventions, and triangulation of the findings reported from population-level measures, *i.e.* folic acid fortification.

Most of the primary studies on anaemia and NTDs were conducted much earlier than the 2010s [36] when a systematic approach to evidence synthesis started to be commonly used in the field of nutrition [37,38]. The evidence on anaemia and NTD consists of RCTs demonstrating consistently significant protective effects. The biological mechanism of the causal role of folate in megaloblastic anaemia has also been well documented [39,40]. The evidence on anaemia and NTD has contributed to the establishment of thresholds for folate deficiency, insufficiency, and estimated average requirement [4,6] and policy actions globally. Real-world data from ecological studies on the effectiveness of folic acid fortification in prevention of NTD in the populations further triangulates the evidence. For example, as of July 2023, a total of 116 countries has legislated mandatory or voluntary folic acid fortification of their staple grain [41]. Mandatory fortification has been demonstrated to have significantly reduced the prevalence of NTD globally [10,41,42]. Considering all these factors, we assessed that the evidence on the relationship between folate and the risk of megaloblastic anaemia and NTD was convincing.

## DISCUSSION

### Summary of findings on anaemia and NTDs

The number of systematic reviews or meta-analyses on folate and haematological indicators including anaemia was limited. However, as folate deficiency has traditionally been diagnosed by the haematologic parameters [2], we did not expect to find recent reviews on this topic.

Earlier evidence from intervention trials and later evidence from retrospective studies both demonstrated that maternal folate intake was significantly inversely associated with the prevalence of NTD at birth. These studies took place in countries in Europe and Africa and had similar ranges of 0.4-5.0 mg/d without further dose-response analyses being available. These findings agreed with evidence examining the folate status as plasma/serum or RBC concentrations.

The importance of folate in prevention of NTD was further supported by ecological studies reporting the effectiveness of mandatory folic acid fortification in reduction of NTD. As some authors [34] noted, prospective studies have been sparse since the start of food fortification in the US and Canada and the more recent retrospective studies reported attenuated or inconsistent effects potentially due to reduced prevalence of folate inadequacy, recall bias or misclassification bias. Among the syntheses on folic acid fortification, only one focusing on four countries in Africa reported the doses used in the fortification. We note that reviews of fortification studies can benefit from more detailed information on the fortification programmes (dose, duration, target grain), study period, and compliance status, which may increase heterogeneity in the identified data.

### Equity and global health in the evidence on anaemia and NTDs

Although some of the evidence on folate and anaemia came from interventions in LMICs, a larger volume of data originated form high-income countries (HICs). Given the early timeframe of the primary research conducted on anaemia, the HIC predominance may be partly stem from a lack of resources and less prioritisation of this outcome in health research in LMICs at the time. A sensitivity analysis at the level of primary studies was out of the scope of this review; however, we noted that the data contributions from the LMICs (risk estimates and variances) were generally comparable in direction and magnitude to those from the HICs and the related pooled estimates.

With regard to NTDs, a substantial number of syntheses examined the relationship predominantly or entirely in LMICs, partly motivated to assess the impact of folic acid fortification. The findings from the LMICs were consistent with the earlier findings in HICs. Indeed, the uptake of folic acid fortification is higher in the LMICs compared to the HICs [43], where recommendations for voluntary fortification are predominant [44]. Several policy assessments and narrative reviews – not eligible for our series – have reported that the fortification policies have been effective in reducing the prevalence of NTDs globally [10,42,45].

### Contributions of the review series

We retrieved a large volume of reviews investigating the potential role of folate in the pathology of many health outcomes, with each evidence reporting on multiple sub-categories of disorders in different groups of individuals.

For this series of umbrella reviews, we clearly delineated the study population for each association identified; grouped folate exposure measures to reduce heterogeneity and misclassification; and considered potential biases arising from component study designs. For example, in addition to the overall pooled estimates, we reported subgroup estimates whenever available (*i.e.* stratification by age groups, geographical regions, exposure measures, dose and durations) to understand the differential effects of folate intake, if any, in different population groups. We conducted sensitivity analyses if the evidence for a unique association was pooled from prospective and retrospective studies. We also modified the credibility assessment criteria to better evaluate synthesis findings that point to non-significant associations in the overall evidence hierarchy. In this way, we triangulated the existing evidence on the relationship between folate status and various health outcomes estimated across different study designs, population subgroups, and measurement methods.

Another focus in this series was to bring a global perspective to the evidence landscape: we reported on countries that contributed to the primary studies as well as the evidence synthesised from regions or subgroups that have not been previously well represented in research.

### Limitations of the review series

Nevertheless, our work across the review series had some limitations. First, as a review of reviews, evidence would only be included if a systematic review on the topic had been completed, therefore not all available evidence (*i.e.* all primary evidence) would have been captured. Also, the most recent studies would be missed if they were not identified in the search of a systematic review. Second, we were limited by incomplete descriptions of study population and exposure/ intervention details. Basic characteristics of component studies (*i.e.* age, sex, country, dose and duration, comparator, thresholds for categorising intake/status) were often incomplete or not reported, which substantially restricted our efforts to provide as detailed and precise a synthesis as possible. Some data (*e.g.* dose and duration of folic acid supplementation) also exhibited very wide ranges. The incomplete or broadly defined information did not influence our selection of evidence, credibility assessment, or overall conclusions, which were based on precision and bias measures. However, we strived to capture and transparently report the data made available by the syntheses to help readers make contextualised understanding of the evidence. This evidence, in its current form may also help with generating hypotheses for further research, particularly in areas where folate research is new or relatively sparse. Third, we observed a substantial level of incomplete reporting of the assays used to measure folate concentration in the component studies. Thresholds for insufficiency vary by the type of assays used [46,47]; however, the authors often did not describe the approach used to harmonise the measurements from different assays used in the component studies or to pool the measurements of different types of blood samples (*i.e.* RBC and serum).

Fourth, there is under-representation of evidence from LMICs. We did not limit the database search by language to gauge the volume of evidence in non-English languages and excluded only eight articles that were not available in English; however, we note that more innovative approaches can be made to search for evidence in non-English-based databases and archives. Lastly, this work was limited to the syntheses published in peer-reviewed journals. Reports from governments or non-governmental organisations with a focus of interest in subgroups of population or sub-categories of disease might have been missed.

We also note that the landscape of evidence and the challenges of synthesising evidence vary somewhat by health outcome category. We plan to discuss these limitations that are more specific to each outcome examined in our subsequent reviews.

## CONCLUSIONS

We introduce a series of umbrella reviews to be presented in five subsequent articles, examining the association between folate and multiple health outcomes in various populations and settings. These reviews bring together the existing evidence on folate presented across 283 systematic review or meta-analyses in a coherent framework, which is then evaluated for credibility using robust criteria.

In anaemia and NTDs, we identified robust evidence supporting the protective role of folate status based on intervention trials and observational studies. These works laid the groundwork for folate-related policy measures worldwide prior to the adoption of systematic methodology to evidence synthesis. More recent reviews examining the role of folate in other less well understood health conditions, where the evidence is not so conclusive, will be presented in the subsequent reports.

## Additional material


Online Supplementary Document
