# Supplementary material for: Folate and global health umbrella review series, part 1: methodological framework and syntheses on anaemia and neural tube defects
Source: J Glob Health. 2026 Jan 30;16:04014. doi: 10.7189/jogh.16.04014 (PMC12856383; doi:10.7189/jogh.16.04014)
Supplement: Online Supplementary Document [file jogh-16-04014-s001.pdf]

Supplement to: Yoo S, Montazeri A, Bennett D, Bo Y, Chen P, Duthie S, Jensen N, Kaminga A, Lai JS, Li X, MacFarlane AJ, Martinez H, McNulty H, Momoli F, Mossey P, Mullie P, Munger R, Parajuli RP, Potvin Kent M, Rubini M, Senekal M, Sikora L, Stintzi A, Theodoratou E, Wang H, Yajnik C, Yaktine A, Little J. Methodological framework and syntheses on anaemia and neural tube defects. J Glob Health. 2026;16:04014.

Table S1. Search strategy

**Medline**
1. exp Folic Acid/
2. ((vitamin* or vit or co?enzym*) adj2 (b9 or b 9 or m)).tw,kw.
3. (folate or folic acid or folacin or folvite or pteroylglutamic acid or acfol or acifolic or acido folico or filicine or folacid or folart or folavit or folavite or foldivie or foliamin or folicid or folicet or folina or folinsyre or folitab or folium acid or folivit or folsan or folsau or folveriam or folvite or ingafol or gravi-fol or lafol or lexpec or megafol or neocepri or pteroyl glutamate or pteroyl monoglutamate or pteroyl monoglutamic acid or rubiefol or unifol).tw,kw.
4. or/1-3
5. diet/ or eating/ or drinking/
6. ((calorie or calories or caloric or diet* or feed* or food* or macronutrient* or micronutrient* or nutrient* or nutritional) adj2 (intake or intakes)).tw,kw.
7. ingest*.tw,kw.
8. Dietary Supplements/
9. ((diet* or food or herbal) adj2 supplement*).tw,kw.
10. (neutraceutical* or nutracentical*).tw,kw.
11. Food Preferences/
12. exp Nutrition Therapy/
13. Foods, Fortified/
14. ((fortified or enriched or supplement*) adj2 food*).tw,kw.
15. Nutritional Status/
16. ((nutrition* or food*) adj2 status*).tw,kw.
17. exp Homocysteine/
18. exp Plasma/
19. Erythrocytes/
20. exp Serum/
21. ((biologic* or clinical or biochemical or serum or immun*) adj2 (marker or markers)).tw,kf.
22. ((end point or end points or endpoint or endpoints) adj surrogate).tw,kf.
23. homocysteine.tw,kf.
24. or/5-23
25. 4 and 24
26. meta-analysis/ or "systematic review"/
27. Systematic Reviews as Topic/
28. (systematic adj2 review*).tw,kw.
29. systematic review.pt.
30. (meta analys* or metaanalys*).tw,kw.
31. meta analysis.pt.
32. (((systematic or state-of-the-art or scoping or literature or umbrella) adj (review* or overview* or assessment*)) or "review* of reviews" or meta-analy* or metaanaly* or ((systematic or evidence) adj1 assess*) or "research evidence" or metasynthe* or meta-synthe*).tw.
33. or/26-32
34. 25 and 33
**Embase**
1. folic acid/
2. ((vitamin* or vit or co?enzym*) adj2 (b9 or b 9 or m)).tw,kw.
3. (folate or folic acid or folacin or folvite or pteroylglutamic acid or acfol or acifolic or acido folico or filicine or folacid or folart or folavit or folavite or foldivie or foliamin or folicid or folicet or folina or folinsyre or folitab or folium acid or folivit or folsan or folsau or folveriam or folvite or ingafol or gravi-fol or lafol or lexpec

or megafol or neocepri or pteroyl glutamate or pteroyl monoglutamate or pteroyl monoglutamic acid or rubiefol or unifol).tw,kw.
4. or/1-3
5. diet/
6. exp food intake/
7. ((calorie or calories or caloric or diet* or feed* or food* or macronutrient* or micronutrient* or nutrient* or nutritional) adj2 (intake or intakes)).tw,kw.
8. ingest*.tw,kw.
9. ingestion/
10. diet supplementation/ or dietary supplement/
11. ((diet* or food or herbal) adj2 supplement*).tw,kw.
12. (neutraceutical* or nutracentical*).tw,kw.
13. food preference/
14. exp diet therapy/
15. fortified food/
16. ((fortified or enriched or supplement*) adj2 food*).tw,kw.
17. nutritional status/
18. ((nutrition* or food*) adj2 status*).tw,kw.
19. homocysteine/
20. exp plasma/
21. erythrocyte/
22. exp serum/
23. ((biologic* or clinical or biochemical or serum or immun*) adj2 (marker or markers)).tw,kf.
24. ((end point or end points or endpoint or endpoints) adj surrogate).tw,kf.
25. homocysteine.tw,kf.
26. or/5-25
27. meta-analysis/ or systematic review/ or systematic reviews as topic/ or meta-analysis as topic/ or "meta analysis (topic)"/ or "systematic review (topic)"/ or exp technology assessment, biomedical/ or network meta-analysis/
28. ((systematic* adj3 (review* or overview*)) or (methodologic* adj3 (review* or overview*))).ti,ab,kf,kw.
29. ((quantitative adj3 (review* or overview* or synthes*)) or (research adj3 (integrati* or overview*))).ti,ab,kf,kw.
30. umbrella review*.ti,ab,kf,kw.
31. ((integrative adj3 (review* or overview*)) or (collaborative adj3 (review* or overview*)) or (pool* adj3 analy*)).ti,ab,kf,kw.
32. or/27-31
33. 4 and 26 and 32

CDSR

1. ((vitamin* or vit or co?enzym*) adj2 (b9 or b 9 or m)).tw,kw.
2. (folate or folic acid or folacin or folvite or pteroylglutamic acid or acfol or acifolic or acido folico or filicine or folacid or folart or folavit or folavite or foldivie or foliamin or folicid or folicet or folina or folinsyre or folitab or folium acid or folivit or folsan or folsau or folveriam or folvite or ingafol or gravi-fol or lafol or lexpec or megafol or neocepri or pteroyl glutamate or pteroyl monoglutamate or pteroyl monoglutamic acid or rubiefol or unifol).tw,kw.
3. or/1-2
4. ((calorie or calories or caloric or diet* or feed* or food* or macronutrient* or micronutrient* or nutrient* or nutritional) adj2 (intake or intakes)).tw,kw.
5. ingest*.tw,kw.
6. ((diet* or food or herbal) adj2 supplement*).tw,kw.
7. (neutraceutical* or nutracentical*).tw,kw.
8. ((fortified or enriched or supplement*) adj2 food*).tw,kw.

9. ((nutrition* or food*) adj2 status*).tw,kw.
10. ((biologic* or clinical or biochemical or serum or immun*) adj2 (marker or markers)).tw,kw.
11. ((end point or end points or endpoint or endpoints) adj surrogate).tw,kw.
12. homocysteine.tw,kw.
13. or/4-12
14. 3 and 13

DARE

1. ((vitamin* or vit or co?enzym*) adj2 (b9 or b 9 or m)).tw,kw.
2. (folate or folic acid or folacin or folvite or pteroylglutamic acid or acfol or acifolic or acido folico or filicine or folacid or folart or folavit or folavite or foldivie or foliamin or folicid or folicet or folina or folinsyre or folitab or folium acid or folivit or folsan or folsau or folveriam or folvite or ingafol or gravi-fol or lafol or lexpec or megafol or neocepri or pteroyl glutamate or pteroyl monoglutamate or pteroyl monoglutamic acid or rubiefol or unifol).tw,kw.
3. or/1-2
4. ((calorie or calories or caloric or diet* or feed* or food* or macronutrient* or micronutrient* or nutrient* or nutritional) adj2 (intake or intakes)).tw,kw.
5. ingest*.tw,kw.
6. ((diet* or food or herbal) adj2 supplement*).tw,kw.
7. (neutraceutical* or nutracentical*).tw,kw.
8. ((fortified or enriched or supplement*) adj2 food*).tw,kw.
9. ((nutrition* or food*) adj2 status*).tw,kw.
10. ((biologic* or clinical or biochemical or serum or immun*) adj2 (marker or markers)).tw,kw.
11. ((end point or end points or endpoint or endpoints) adj surrogate).tw,kw.
12. homocysteine.tw,kw.
13. or/4-12
14. 3 and 13

CINAHL

(MH "Diet+")
(MH "Eating")
(MH "Fluid Intake") OR (MH "Food Intake+") OR (MH "Dietary Reference Intakes")
(MH "Dietary Supplements+") OR (MH "Food, Fortified") OR (MH "Nutrients+")
(MH "Nutritional Status")
(MH "Food Preferences")
(MH "Diet Therapy+")
((calorie or calories or caloric or diet* or feed* or food* or macronutrient* or micronutrient* or nutrient* or nutritional) N2 (intake or intakes))
ingest*
((diet* or food or herbal) N2 supplement)
(neutraceutical* or nutracentical*)
((fortified or enriched or supplement*) N2 food*)
((nutrition* or food*) N2 status*)
(MH "Homocysteine")
(MH "Plasma+") OR (MH "Serum")
(MH "Erythrocytes+")
((biologic* or clinical or biochemical or serum or immun*) N2 (marker or markers))
((end point or end points or endpoint or endpoints) N1 surrogate)
homocysteine
S1 OR S2 OR S3 OR S4 OR S5 OR S6 OR S7 OR S8 OR S9 OR S10 OR S11 OR S12 OR S13 OR S14 OR S15 OR S16 OR S17 OR S18 OR S19
(MH "Folic Acid+")
((vitamin* or vit or co?enzym*) N2 (b9 or b 9 or m))

(folate or folic acid or folacin or folvite or pteroylglutamic acid or acfol or acifolic or acido folico or filicine or folacid or folart or folavit or folavite or foldivie or foliamin or folicid or folicet or folina or folinsyre or folitab or folium acid or folivit or folsan or folsau or folveriam or folvite or ingafol or gravi-fol or lafol or lexpec or megafol or neocepri or pteroyl glutamate or pteroyl monoglutamate or pteroyl monoglutamic acid or rubiefol or unifol)
S21 OR S22 OR S23
S20 AND S24
(MH "meta analysis" OR MH "systematic review" OR MH "Technology, Medical/EV" OR PT "systematic review" OR PT "meta analysis" OR (((TI systematic* OR AB systematic*) N3 ((TI review* OR AB review*) OR (TI overview* OR AB overview*))) OR ((TI methodologic* OR AB methodologic*) N3 ((TI review* OR AB review*) OR (TI overview* OR AB overview*)))) OR (((TI quantitative OR AB quantitative) N3 ((TI review* OR AB review*) OR (TI overview* OR AB overview*) OR (TI synthes* OR AB synthes*))) OR ((TI research ...
S25 AND S26

Table S2a. Characteristics of the evidence syntheses examining the relationship between folate intake/status and anemia

Author (year)	Synthesis type	Study population	Exposure	Outcome	No. of studies (design)	Total (Case)	Country	Comparators
**Anemia**								
Fishman et al. (2000)	SR	Pregnant women	Supplement (0.5-5mg/d for 4-24 weeks)	Mean hemoglobin	11 intervention trials	3,993 (NR)	Myanmar, Thailand, India, Nigeria, Liberia, Australia, South Africa	No or other nutrients
		Pregnant women	Supplement (0.3-1 mg/d for 4-16 weeks)	Megaloblastic anemia	2 RCTs	346 (NR)	Australia, Nigeria	Placebo
		Non-pregnant women and adolescent girls	Supplement (1-15 mg/d for 5-22 weeks)	Mean hemoglobin	4 intervention trials	1,053 (NR)	USA, Thailand, Malaysia	Other nutrients
		Premature and low birth weight infants	Supplement (0.05-0.1 mg/d for 4 weeks – 12 months)	Megaloblastic anemia	5 intervention trials	700 (NR)	UK, USA	Placebo
Lassi et al. (2013)	MA	Pregnant women	Supplement (0.45-5 mg/d)	Pre-delivery anemia	8 RCTs	4,149 (577)	UK, Nigeria, India, Myanmar, Australia	None or placebo or other nutrients
		Pregnant women	Supplement (0.05-350 mg/d)	Pre-delivery hemoglobin	12 RCTs	1,806 (NR)	UK, Switzerland, France, Finland, Chile, South Africa, Nigeria, Thailand	None or placebo or other nutrients
		Pregnant women	Supplement (0.01-5mg/d)	Megaloblastic anemia	4 RCTs	3,839 (89)	UK, Nigeria	None or placebo or other nutrients

MA: meta-analysis; NR: not reported; RBC: red blood cell; RCT: randomized controlled trial; SR: systematic review

Table S2b. Characteristics of the evidence syntheses examining the relationship between folate intake/status and neural tube defects (NTDs)

Author (year)	Synthesis type	Study population	Exposure	Outcome	No. of studies (design)	Total (Case)	Country	Comparators
Wolff et al. (2009)	SR	Pregnant women	Maternal supplement (1 PC: 0.8 mg/d for 1 month)	NTD	3 (1 PC, 2 CC)	7,628 (718)	US, Hungary	None or placebo
Blencowe et al. (2010)	MA	Pregnant women with previously affected pregnancies	Maternal supplement (0.36 mg/d or 5.0 mg/week)	NTD recurrence	3 RCT	NR (NR)	UK, Ireland, Hungary, Australia, Italy, France, Canada, Russia	None or placebo
		Pregnant women	Maternal supplement (0.36 mg/d or 5.0 mg/week)	NTD	4 (1 RCT, 3 PC)	NR (NR)	Hungary, China	None or placebo
		Pregnant women	FA fortification (NR)	NTD	8 Before-After	NR (NR)	Chile, South Africa, Argentina, USA, Canada	Pre-fortification vs. post-fortification
De-Regil et al. (2010)	MA	Women who were < 12 weeks pregnant at the time of intervention	Maternal supplement (FA only) (0.36-4.0 mg/d)	NTD	2 intervention trials	299 (9)	Ireland, UK	None or placebo
			Maternal supplement (FA with other micronutrients) (0.36-4.0 mg/d)	NTD	4 intervention trials	5,806 (48)	Hungary, Israel, Australia, Canada, Russia, France, UK, Ireland, India	Other micronutrients
Imdad et al. (2011)	MA	Pregnant women	Maternal supplement (NR)	NTD	4 (1 RCT, 3 PC)	NR (NR)	NR	None or placebo
		Pregnant women	Maternal supplement (NR)	NTD recurrence	3 RCT	NR (NR)	NR	None or placebo
		Pregnant women	FA fortification (NR)	NTD	11 Before-After	NR (NR)	NR	Pre-fortification vs. post-fortification
Ramakrishnan et al. (2012)	SR	Pregnant women	Maternal supplement (0.4-5.0 mg/d)	NTD	10 (5 RCT, 2 community-based intervention, 3 CC)	322,909 (NR)	Hungary, UK, Israel, Australia, Canada, Russia, France, India, China, US	None or placebo

De-Regil et al. (2015)	MA	Women who were < 12 weeks pregnant at the time of intervention	Maternal supplement (FA with other micronutrients) (0.36-4.0 mg/d)	NTD	4 intervention trials	6,512 (49)	Hungary, Israel, Australia, Canada, Russia, France, UK, Ireland, India	Other micronutrients
					2 intervention trials	1,371 (28)	Ireland, UK, Israel, Australia, Canada, Russia, France	Same other nutrients
Tang et al. (2015)	MA	Pregnant women (case: 24-30.5 yrs, control: 26.5-37.8 yrs)	Plasma/serum	NTD	15 PC	3,124 (869)	Netherlands, China, Ireland, USA, Turkey, Iran, Finland, UK, Canada, Egypt	Mothers of unaffected infants
	MA	Pregnant women (case: 24-42.9 yrs, control: 25.6-39 yrs)	Plasma/serum	NTD	14 CC	2,260 (825)	Africa, Netherlands, UK, Mexico, Canada, Brazil, USA, India, Egypt, Norway	Mothers of unaffected infants
	MA	Pregnant women (case: 30.5 yrs, control: 37.8 yrs)	RBC	NTD	14 PC	607 (214)	Netherlands, Ireland, Mexico, UK	Mothers of unaffected infants
	MA	Pregnant women (case: 26.5-42.9 yrs, control: 26.9-35.6 yrs)	RBC	NTD	10 CC	848 (414)	Africa, Netherlands, UK, Mexico, Canada, Norway, India, USA	Mothers of unaffected infants
Viswanathan et al. (2017)	SR	Pregnant women	Maternal supplement (NR)	NTD	7 RCT	4,862 (NR)	Hungary	None or placebo or supplementation without folic acid
		Pregnant women		NTD	3 PC	19,982 (NR)	Hungary, US	
		Pregnant women		NTD	8 CC	28,880 (NR)	US	
Bitwew et al. (2020)	MA	Newborns	Maternal supplement (NR)	NTD	4 CC	1,592 (418)	Ethiopia	Mothers of unaffected infants
Atlaw et al. (2021)	MA	Newborns	Maternal supplement (NR)	NTD	7 (6 CC, 1 CS)	1,963 (463)	Africa (Egypt, Tunisia, Ethiopia, Algeria)	Mothers of unaffected infants
Lassi et al. (2021)	MA	Pregnant women aged 16-49 yrs	Maternal supplement (0.4-5.0 mg/d)	NTD	2 intervention trials	248,056 (130,243)	China, Honduras, Brazil, Cuba	Placebo
Yadav et al. (2021)	MA	Pregnant women	Serum/RBC	NTD	36 CC	6,114 (2,131)	NR	Mothers of unaffected infants
Castillo-Lancellotti et al. (2013)	SR	Pregnant women	FA fortification of flour in 2005-2009 (NR)	NTD	27 (Before-After)	NR (NR)	Chile, Argentina, Brazil, Canada, Costa Rica, Iran, Jordan, South Africa, USA	Pre-fortification vs. post-fortification

Atta et al. (2016)	MA	Birth cohorts	FA fortification (NR)	Spina bifida	123 population-based studies	NR (NR)	NR	Mandatory vs. no fortification
Keats et al. (2019)	MA	Live and Stillbirths in LMIC **	FA fortification (wheat flour fortified with FA 1.5-5.0 mg/kg, maize flour fortified with FA 1.3-2.2 mg/kg)	NTD	17 Mixed *	19,816,008 (13,494)	Fiji, Cameron, Iran, Brazil, South Africa, Peru, Costa Rica, Argentina, Tanzania, Jordan, China	Pre-fortification vs. post-fortification
				Spina bifida	9 Mixed *	21,175,429 (6,385)		
				Anencephaly	9 Mixed *	21,174,429 (6,876)		
				Encephalocele	8 Mixed * 21,049,821	21,049,821 (1,857)		

* Mixed: combination of multiple study designs (hospital-based surveillance, repeated cross-sectional, before-after studies, retrospective administrative records)

** LMIC countries studied include Fiji, Cameroon, Iran, Brazil, South Africa, Peru, Costa Rica, Argentina, Tanzania, Jordan, China

CC: case-control; CS: cross-sectional; FA: folic acid; LMIC: low- and middle-income countries; MA: meta-analysis; NR: not reported; NTD: neural tube defects; PC: prospective cohort; RBC: red blood cell; RCT: randomized controlled trial; SR: systematic review

Table S3. Summary of the meta-analyses reporting the association of folate intake/status with the risk of neural tube defects (NTDs)

Author (year)	Exposure	Country/Region	No. studies (design)	No. total (case)	Comparator	Summary effect	I^2	P Egger
**Prevalence of NTD at birth**								
De-Regil et al. (2010)	Maternal supplement (FA only) (0.36-4.0 mg/d)	Ireland, UK	2 intervention trials	299 (9)	None or placebo	RR=0.32 (0.08, 1.34)	0% (p=0.60)	
	Maternal supplement (FA with other nutrients) (0.36-4.0 mg/d)	Hungary, Israel, Australia, Italy, France, Canada, Russia, UK, Ireland, India	4 intervention trials	5,806 (48)	Other nutrients	RR=0.29 (0.15, 0.56)	0% (p=0.74)	
Blencowe et al. (2010)	Maternal supplement (0.36 mg/d or 5.0 mg/week)	Hungary, China	4 (1 RCT, 3 PC)	NR (NR)	None or placebo	RR=0.38 (0.29, 0.51)	27.9% (p=0.24)	
Imdad et al. (2011)	Maternal supplement (NR)	NR	4 (1 RCT, 3 PC)	NR (NR)	None or placebo	RR=0.38 (0.29, 0.51)		
De-Regil et al. (2015)	Maternal supplement (FA with other nutrients vs other nutrients) (0.36-4.0 mg/d)	Hungary, Israel, Australia, Canada, Russia, France, UK, Ireland, India	4 intervention trials	6,512 (49)	Other micronutrients	RR=0.31 (0.16, 0.60)	0% (p=0.74)	
	Maternal supplement (FA with other nutrients vs same other nutrients) (0.36-4.0 mg/d) NTD	Ireland, UK, Israel, Australia, Canada, Russia, France	2 intervention trials	1,371 (28)	Same other nutrients	RR=0.29 (0.12, 0.70)	0% (p=0.92)	
Bitwew et al. (2020)	Maternal supplement NTD	Ethiopia	4 CC	1,592 (418)	Unaffected births	OR=0.32 (0.17, 0.60)	36% (p=0.19)	
Atlaw et al. (2021)	Maternal supplement (NR) NTD	Africa (Egypt, Tunisia, Ethiopia, Algeria)	7 (6 CC, 1 CS)	1,963 (463)	Unaffected births	OR=0.4 (0.19, 0.85)	78% (p<0.001)	
Lassi et al. (2021)	Maternal supplement (0.4-5.0 mg/d) NTD	China, Honduras, Brazil, Cuba	2 intervention trials	248,056 (130,243)	Placebo	RR=0.53 (0.41, 0.67)	0% (p=0.36)	
Tang et al. (2015)	Plasma/serum folate	See below	29 (14 CC, 15 PC)	5,384 (1,694)	Unaffected mothers	RoM=0.93 (0.88, 0.97)	73%	p=0.05
		Africa, Netherlands, Britain, Mexico, Canada, Brazil,	14 (CC)	2,260 (825)		RoM=0.95 (0.86, 1.04)	84%	

		America, India, Egypt, Norway						
		Netherlands, China, Ireland, America, Turkey, Iran, Finland, Britain, Canada, Egypt	15 (PC)	3,124 (869)		RoM=0.91 (0.86, 0.96)	40%	
		NR	Asian (NR)	1,790 (699)		RoM=0.88 (0.81, 0.90)	2%	
		NR	*Caucasian* (NR)	2,813 (666)		RoM=0.93 (0.88, 0.99)	67%	
Tang et al. (2015)	RBC folate	See below	24 (10 CC, 14 PC)	1,455 (628)	Unaffected mothers	RoM=0.92 (0.86, 0.98)	72%	p=0.22
		Africa, Netherlands, Britain, Mexico, Canada, Norway, India, America	10 CC	848 (414)		RoM=0.91 (0.84, 0.98)	74%	
		Netherlands, Ireland, Mexico, UK	14 PC	607 (214)		RoM=0.95 (0.80, 1.14)	67%	
		NR	Asian (NR)	59 (35)		RoM=0.74 (0.55, 0.98)	NA	
		NR	*Caucasian* (NR)	1,032 (405)		RoM=0.90 (0.82, 0.98)	78%	
Yadav et al. (2021)	Serum/RBC	NR	36 CC	6,114 (2,131)	Unaffected births	SMD -0.48 (-0.77, -0.19)	95.73% (p<0.001)	p>0.05
			Asian (NR)	Asian (NR)		SMD=-1.37 (-2.41, -0.61)	97.85% (p<0.001)	
			Caucasian (NR)	*Caucasian* (NR)		SMD=-0.17 (-0.35, 0.004)	78.89% (p<0.001)	
			African (NR)	African (NR)		SMD=-0.03 (-0.56, 0.49)	60.89% (p=0.11)	
Blencowe et al. (2010)	Fortification<br><br>(NR)	Hungary, China	8 Before-after	NR (NR)	Pre-fortification vs. post-fortification	RR=0.54 (0.46, 0.63)	69.2% (p=0.002)	
Keats et al. (2019)	Fortification (wheat 1.5-5.0 mg/kg; maize 1.3-2.2 mg/kg)	LMICs**	17 Mixed *	19,816,008 (13,494)	Pre-fortification vs. post-fortification	OR=0.59 (0.49, 0.70)	84% (p<0.00001)	

**Spina bifida**								
Atta et al. (2016)	Fortification (NR)	NR	123 Before-after	NR (NR)	Mandatory vs no fortification	Among live births, 33.86 per 100,000 (31,05, 36.92) among mandatory fortification countries vs 48.35 per 100,000 (41.07, 56.93) among voluntary fortification or no fortification countries.<br>Among live births and stillbirths, 30.37 per 100,000 (27.42, 33.63) mandatory fortification countries vs 47.74 (43.66, 52.20) among voluntary fortification or no fortification countries.<br>Among live births, still births, terminated pregnancies, 35.22 per 100,000 (32.18, 38.56) vs 52.29 per 100,000 (46.28, 59.08) among voluntary fortification or no fortification countries.		
Keats et al. (2019)	Fortification (wheat 1.5-5.0 mg/kg; maize 1.3-2.2 mg/kg)	LMICs**	9 Mixed *	21,175,429 (6,385)	Pre-fortification vs. post-fortification	OR=0.66 (0.53, 0.82)	88% (p<0.00001)	
**Anencephaly**								
Keats et al. (2019)	Fortification (wheat 1.5-5.0 mg/kg; maize 1.3-2.2 mg/kg)	LMICs**	9 Mixed *	21,174,429 (6,876)	Pre-fortification vs. post-fortification	OR=0.49 (0.40, 0.60)	78% (p<0.0001)	
**Encephalocele**								
Keats et al. (2019)	Fortification (wheat 1.5-5.0 mg/kg; maize 1.3-2.2 mg/kg)	LMICs**	8 Mixed *	21,049,821 (1,857)	Pre-fortification vs. post-fortification	OR=0.64 (0.47, 0.88)	75% (p=0.0003)	
**NTD recurrence**								
Blencowe et al. (2010)	Maternal supplement (0.36 mg/d or 5 mg/week)	UK, Ireland, Hungary, Australia, Italy, France, Canada, Russia	3 RCT	NR (NR)	None or placebo	RR=0.30 (0.14, 0.65)	0% (p=0.87)	
Imdad et al. (2011)	Maternal supplement (NR)	NR	3 RCT	NR (NR)	None or placebo	RR=0.30 (0.14, 0.65)	0%	

* Mixed: combination of multiple study designs (hospital-based surveillance, repeated cross-sectional, before-after studies, retrospective administrative records)

** LMICs: Fiji, Cameroon, Iran, Brazil, South Africa, Peru, Costa Rica, Argentina, Tanzania, Jordan, China

Caucasian is italicized as it is an imprecise and outdated terminology which is commonly used to describe individuals of European decent

CC: case-control study; CCoh: Case cohort; CS: Cross sectional; LMIC: low- and middle-income country; NCC: nested case-control; NR: not reported; PC: prospective cohort; RBC: red blood cell; RoM: ratio of means
